# Biological Control of Stem Rot of Groundnut Induced by *Sclerotium rolfsii* sacc.

**DOI:** 10.3390/pathogens13080632

**Published:** 2024-07-28

**Authors:** Prabhu Narayan Meena, Ashok Kumar Meena, Rahul Kumar Tiwari, Milan Kumar Lal, Ravinder Kumar

**Affiliations:** 1 ICAR—National Research Centre for Integrated Pest Management, Indian Agricultural Research Institute, New Delhi 110012, India; 2 ICAR—Central Research Institute for Jute and Allied Fibres, Kolkata 700120, India; 3 Department of Plant Pathology, College of Agriculture, Swami Keshwanand Rajasthan Agricultural University, Bikaner 334006, India; akmeenapathology@gmail.com; 4 ICAR—Indian Institute of Sugarcane Research, Lucknow 226002, India; rahultiwari226@gmail.com; 5 ICAR—National Rice Research Institute, Cuttack 753006, India; milan2925@gmail.com; 6 ICAR—Indian Agricultural Research Institute, New Delhi 110012, India

**Keywords:** *Arachis hypogaea*, *Trichoderma viride*, *Trichoderma harzianum*, *Pseudomonas fluorescens*, *Bacillus subtilis*

## Abstract

Stem rot of groundnut (*Arachis hypogaea* L.) caused by *Scelrotium rolfsii* is the main threat to groundnut production, causing significant economic losses. The present study aims to provide an overview of the potentiality of *Trichoderma viride* (Tv), *Trichoderma harzianum* (Th), *Pseudomonas fluorescens* (Pf), and *Bacillus subtilis* (Bs), applied either individually or in mixed combination, against *Sclerotium rolfsii* (isolate SrBKN). The treatment with *T. harzianum* and *P. fluorescens* resulted in the highest mycelial growth inhibition (79.61, 83.51, and 86.77%), followed by *T. viride* and *P. fluorescens* (75.74, 79.63, and 83.14%). Under in vitro conditions, the combination of bio-agents at 5% culture filtrate proved to be superior against the test pathogen. Seed treatment and soil application of *T. harzianum* and *Pf* at 10 (5 + 5) g kg^−1^ + 10 (5 + 5) kg ha^−1^, followed by seed treatment and soil application of *T. viride* and *Pf* at 10 (5 + 5) g kg^−1^ + 10 (5 + 5) kg ha^−1^, resulted in the lowest disease incidence (7.40 and 8.0%), highest disease control (69.37 and 66.88%), maximum dry weight (151 and 147 g plant^−1^), highest increase in dry weight (75.58 and 70.93), highest pod yield (2665 and 2498 kg ha^−1^), and highest increase in pod yield (96.38 and 84.08%) under in vivo conditions. We present an effective bio-control-based management module from the lab to the field for the successful control of groundnut stem rot caused by *S. rolfsii*. Based on the results, it is concluded that the dual formulation of *T. harzianum* and *P. fluorescens*, followed by *T. viride* and *P. fluorescens*, were the most effective BCAs in suppressing the *S. rolfsii*. Therefore, an integrated disease management module with these BCAs needs to be developed and validated with a farmers’ participatory mode under field conditions.

## 1. Introduction

Groundnut (*Arachis hypogaea* L.), commonly called peanut, goober pea, pindad jack nut, and monkey nut, is a major oilseed crop cultivated in southern and northern India. It is also known as the king of oilseeds and was cultivated as early as 1000 B.C. [1]. India occupies the second position after China in terms of area and production of groundnut; it is chiefly cultivated in Gujarat, Rajasthan, A.P., Tamil Nadu, and Karnataka. It is grown in a 5.31 million ha area with an annual production of 7.57 million tons and productivity of 1424 kg ha^−1^ in India [2].

The groundnut crop is prone to various biotic stresses induced by bacterial, fungal, and viral diseases. Among fungal diseases, *Sclerotium rolfsii* is a deadly white mold that is soil-borne and has a wide host range [3]. Higher temperatures and moisture are favorable conditions for the development and survival of *S. rolfsii*. The incidence of *S. rolfsii* typically appears in one-month-old crops in which mycelium covers the stem near the soil surface, producing organic acids that are harmful to plants and cause cell necrosis. The primary diagnostic symptoms are mycelium formation and sclerotia on diseased tissue [4]. According to Akgul et al. 2011 [5], stem rot causes a 27% loss of groundnut crops in India, 10–50% in South America, and more than 25% in Australia [5]. The loss rate in severely infected fields can reach 80% [6,7]. Stem rot is most common during the *kharif* season, alone or in combination with other diseases, and it produces significant crop yield losses. This disease is the most devastating, ubiquitous, and yield-reducing factor in groundnut crops, particularly in Rajasthan’s northwestern plain zone [8].

Chemical pesticides play a crucial role in modern agricultural developments by reducing agricultural pests, and these chemicals are still growers’ first choice for effectively and rapidly controlling fungal diseases. However, frequent and indiscriminate use of these chemical pesticides causes various ecological disruptions, human health risks, a decrease in the population of beneficial microorganisms in the soil, ozone layer depletion, and the development of pesticide resistance [9,10]. Fungicides presently recommended to control this disease are protected for a limited period. Cultural methods (rouging, avoiding plant injury, and eradication of weed host) of stem rot management are not sufficiently effective due to the prolonged soil-borne nature of *S. rolfsii*. The most effective approach is to use resistant cultivars; however, availability and durability are important difficulties. In this context, biological controls are considered promising alternatives to fungicides and have opened up new vistas in stem rot management. Biological control agents (BCAs) have unique action mechanisms, including hyperparasitism, competition, antibiosis, and induced systemic resistance [11]. Studies conducted by Elad et al. (1983), Rekha et al. (2012), Safari Motlagh et al. (2022), and F. Sultana and M. M. Hossain (2022) have shown the efficacy of biological control agents against *Rhizoctonia*, *Pythium*, *Fusarium*, *Verticillium*, and *Sclerotium rolfsii* under in vitro and in vivo conditions [12,13,14,15]. The need for bio-agents in India and the world is continuously increasing due to the adverse effects of chemical pesticides on the environment [16,17]. When BCA-based formulation is used for stem rot management, its efficacy can be equal to or better than traditional fungicides in groundnut crops due to chemical compatibility [18]. The combination of bio-agents and lower doses of fungicides has been utilized to combat various soil-borne diseases [19].

The primary goals of this study are to develop a dual microbial consortium and to investigate the potential of antagonistic BCAs in terms of efficiency and efficacy in reducing mycelial growth and disease incidence of stem rot under field conditions, as well as their effects on dry weight and pod yield of groundnut plants.

## 2. Materials and Methods

### 2.1. Isolation and Identification of Causal Pathogen

**Figure 1:**
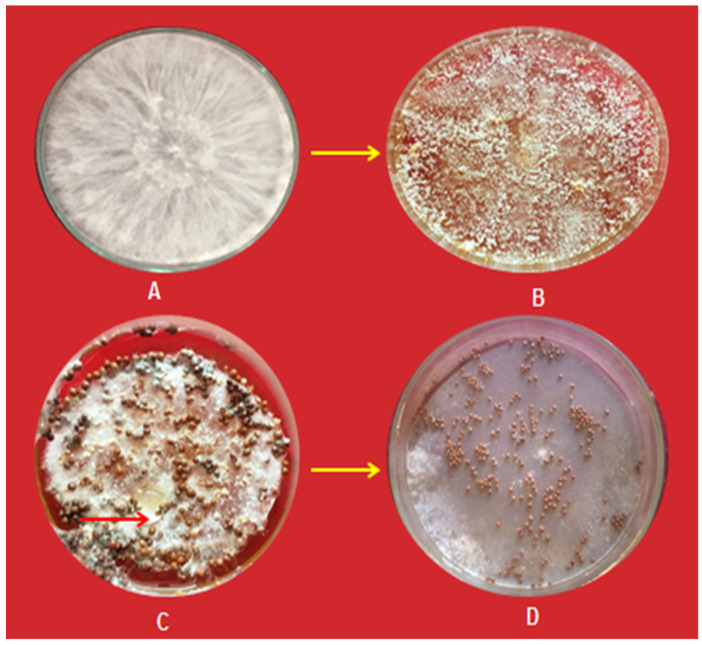
Different sclerotium stages of *Sclerotium rolfsii*. (**A**) White fluffy growth of mycelium; (**B**) Young sclerotia; (**C**) Gummy material around sclerotia; (**D**) Mature sclerotia.

**Figure 2:**
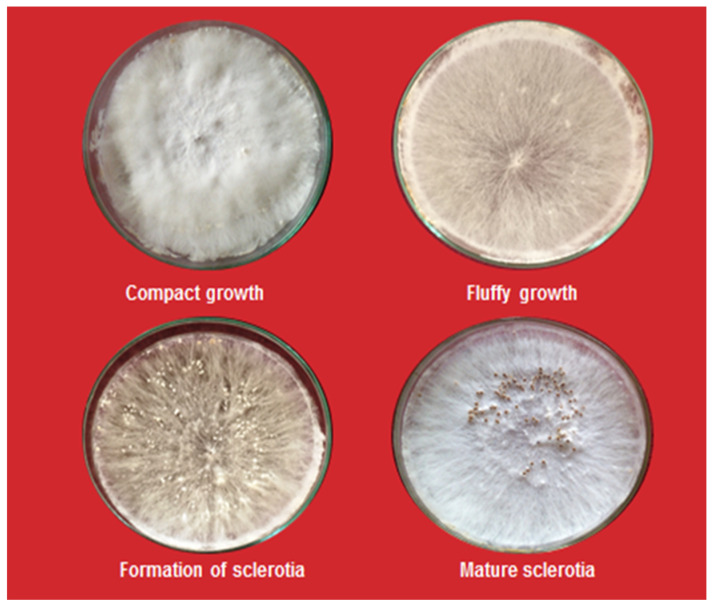
Identification of *Sclerotium rolfsii* on the basis of morphological features.

Stem rot-infected groundnut plants showing symptoms of disease and sclerotia were collected from the Bikaner (isolate SrBKN), Jaipur (isolate SrJPP, Churu (isolate SrC), and Sikar (isolate SrS) districts of Rajasthan, India. Infected samples were gently washed in tap water and subsequently with sterile water and cut into small pieces, then sterilized with one percent NaOCl solution for 1 min. After three washings, diseased stem pieces were aseptically transferred into streptomycin sulfate (0.003%/L) mixed potato dextrose agar (PDA) media contained in Petri dishes. Then, plates were incubated in BOD for seven days at 26 ± 2 °C for the growth of the pathogen. The mycelium developed from diseased tissues was sub-cultured aseptically into PDA tubes and Petri plates. The local isolate SrBKN of *Sclerotium rolfsii* was used in this study, which was purified by the hyphal tip technique [20]. *S. rolfsii* was identified based on morphological and cultural attributes of mycelium growth appearance and sclerotia. The morphological characters, viz. sclerotium formation, their color, shape, size, and mycelium growth were recognized under low power magnification (10×) microscope and identified as per key points described in “Illustrated Genera of Imperfect Fungi” [21], Figure 1 and Figure 2. The isolate SrBKN of *S. rolfsii* was selected from our previous published work after confirmation by both morphological and molecular basis using ITS primers [22].

### 2.2. Pathogenicity Assay and Validation of Koch’s Postulate

The pathogenicity test of *S. rolfsii* causing stem rot disease in groundnuts was confirmed in earthen pots by the stem inoculation technique described by [23]. Sterilized earthen pots (24 cm diameters) were filled with autoclaved, sterilized soil, then seeds of groundnut (HNG-10) were surface sterilized with 5% (*v*/*v*) sodium hypochlorite for 5 min followed by three washings with water and then sown in the earthen pots. After germination, 4–5 seedlings were maintained in each pot for inoculations of the test pathogen. For inoculum, sand maize meal medium was prepared in 2:1 proportion, suitably moistened, and transferred in 250 mL Erlenmeyer flasks, sterilized at 15 psi for 30 min. Fresh culture of *S. rolfsii* was inoculated in sand maize meal medium and upheld at 28 ± 2 °C for 10 days. The inoculum of *S. rolfsii* was added to 20 g kg^−1^ soil near the stem zone and covered the inoculated area with leaf debris. Pots were regularly watered to keep a suitable moisture regime. Pots without inoculum served as a control. The appearance of disease symptoms on the stems of seedlings and their mortality were recorded periodically. Infected seedling stems were taken to re-isolate the pathogen on PDA media and compared with original isolates of *S. rolfsii* to prove Koch’s postulate theory.

### 2.3. Collection and Maintenance of Bio-Agents

The cultures of *Trichoderma viride*, *Trichoderma harzianum*, *Pseudomonas fluorescens*, and *Bacillus subtilis*, available in the advanced bio-control laboratory, Division of Plant Pathology, College of Agriculture, Bikaner, India, were used in the present study. *Trichoderma* spp. was maintained on 2% (*w*/*v*) PDA, while *Bacillus subtilis* and *Pseudomonas fluorescens* were maintained on nutrient agar (NA) media. All the freshly grown bio-agents were incubated in biological oxygen demand (BOD) at 28 ± 1 °C temperature for use in a further study.

### 2.4. In Vitro Antagonistic Assay

The dual culture method was followed to ascertain the antagonistic activity of *T. viride* and *T. harzianum*. Mycelial discs (5 mm diameter) of the pathogen and each bio-agent were kept on the surface of Petri plates containing potato dextrose agar media at 5 cm apart from the center. Control was maintained only with the mycelial disc of the pathogen in Petri dish. Petri dishes were incubated at 28 ± 2 °C for 7 days to grow the pathogen and bio-agents. The mycelial growth inhibition of fungus was calculated by using the formula suggested by Vincent [24]; Percent inhibition = Mycelial growth (mm) of *S. rolfsii* in control—Mycelial growth (mm) of *S. rolfsii* in the presence of fungal antagonist/Mycelial growth (mm) of *S. rolfsii* in control × 100.

The efficacy of bacterial antagonists *P. fluorescens* and *B. subtilis* on *S. rolfsii* was determined following the paper disc method [25]. *P. fluorescens* and *B. subtilis* were grown on Pseudomonas agar media and Nutrient agar media (PAF and NA) in test tube slants and incubated at 27 °C and 25 °C for 48 h, respectively. Ten millilitres of sterilized water was added into each bacterial bio-agent slant. The circular discs (5 mm in dia.) of the Whatman filter (No. 42) were cut and, after dipping in the suspension of respective bacterial antagonists, placed 1 cm inward from the periphery of Petri dishes containing potato dextrose agar media at four equal distances, having in the center the inoculum of the pathogen (*S. rolfsii*). The inoculated dishes were placed in an incubator at 28 ± 2 °C for 24 h and observations were recorded after incubation. The mycelial growth inhibition of *S. rolfsii* by respective bio-agents was estimated by using the following formula: Percent inhibition = Mycelial growth (mm) of *S. rolfsii* in control—Mycelial growth (mm) of *S. rolfsii* in the presence of bacterial antagonist/Mycelial growth (mm) of *S. rolfsii* in control × 100.

The effect of different culture filtrates of bio-agents, either alone or with combinations, was also tested on the pathogen [26]. Under culture filtrate test, experiments were comprised with nine treatments: T_1_—*T. viride*; T_2_—*T. harzianum*; T_3_—*B. subtilis*; T_4_—*P. fluorescens*; T_5_—*T. viride* + *P. fluorescens*; T_6_—*T. harzianum* + *P. fluorescens*; T_7_—*T. viride* + *B. subtilis*; T_8_—*T. harzianum* + *B. subtilis*; T_9_—Control (without culture filtrate). *T. harzianum* (Th-BKN) and *T. viride* (Tv-BKN) were grown in liquid Potato Dextrose Broth media (PDB), while Nutrient Broth (NB) was used for *P. fluorescens* (Pf-BKN) and *B. subtilis* (Bs-BKN). Bio-agents were incubated in BOD at 28 ± 2 °C for 7 days. The culture of *T. harzianum* (Th-BKN) and *T. viride* (Tv-BKN) was first filtered through double-layered cheese cloth, followed by filtering through (Whatman filter No. 1) filter paper. Then, the culture filtrate of the respective fungal antagonist was centrifuged at 10,000 rpm for 15 min at 4 °C. The centrifuged supernatant was again filtered through a 0.45 µm membrane filter to obtain conidia and fungal fragments free supernatant. The supernatant was stored in the refrigerator. Similarly, *P. fluorescens* (Pf-BKN) and *B. subtilis* (Bs-BKN) bacteria grown in NB media were then centrifuged at 10,000 rpm for 15 min at 4 °C and supernatant was passed through a bacteria-proof filter and stored in a refrigerator for further investigation. The respective supernatant of antagonists was dissolved in PDA medium at 1, 2.5, and 5% concentrations during pouring of the media in Petri dishes to ascertain inhibition potentiality. A mycelial disc (5 mm diameter) of *S. rolfsii* was placed in the center of Petri dishes. Each treatment was replicated thrice. Observation of the mycelial growth of the fungus was recorded after 7 days. Mycelial growth inhibition of fungus in each treatment was calculated according to [27]. Percent growth inhibition (PGI) = [Mycelial growth of *S. rolfsii* in control (mm)—Mycelial growth of *S. rolfsii* in the presence of supernatant (culture filtrate) (mm)/Mycelial growth of *S. rolfsii* in control (mm)] × 100.

### 2.5. Management of S. rolfsii by Bio-Agents under In Vivo Conditions

Pot experiments were conducted in control glasshouse conditions to ascertain the antagonistic activity of bio-agents against *S. rolfsii*; then, these bio-agents were further used in field experiments. The field experiments were conducted to manage the stem rot through seed and soil treatment by bio-agents during *Kharif* of 2017 and 2018 at an instructional research farm (situated at a latitude of 28°01′ N, longitude 73°22′ E and an elevation of 234.7 m above mean sea level). College of Agriculture, Swami Keshwanand Rajasthan Agricultural University, Bikaner falls in tropical climate conditions with an average annual rainfall of 260 mm that are favorable for the perpetuation and development of *S. rolfsii*.

All thirteen treatments, including control, were tested in three replications followed by Randomized Block Design [28] with plot size 3 × 4 m row-to-row spacing of 30 cm and plant-to-plant 10 cm at a fixed site (Table 1). HNG-10 susceptible variety was used in field trials and standard agronomic practices were followed. Talc-based formulations of four antagonists’, viz. *T. harzianum* (Th-BKN), *T. viride* (Tv-BKN), *P. fluorescens* (Pf-BKN), and *B. subtilis* (Bs-BKN), were used at 10 g kg^−1^ seed in individual bio-agent or combination of both bio-agents (5 + 5 g) for seed treatment. In contrast, these were used at 10 (5 + 5) kg ha^−1^ for soil application. Seeds were mixed separately with the respective doses of each bio-agent in a glass beaker for 10–20 min to ensure uniform coating. Farmyard manure (FYM) at 10 t ha^−1^ was equally applied in the field. Seeds were sown in the second week of June. The recommended dose of fertilizer (20 kg N and 40 kg P) was applied 35–40 days after sowing, while 250 kg Gypsum ha^−1^ was given at the peg formation stage. Five irrigations were given at the pegging, flowering, and pod development stage of the crop, and weeding and thinning of plants were done as and when required. Two drenchings of chlorpyriphos at 2.4 l ha^−1^ were applied for termite control.

To maintain the optimum inoculum population, *S. rolfsii* was multiplied on a medium of sand maize (2:1). Flasks containing sterilized media were inoculated with *S. rolfsii* culture and incubated at 26 °C for 15 days. Sand maize meal inoculant of *S. rolfsii* was applied at 50 g per plot and mixed correctly on the top surface of the soil using a hand rack. Similarly, disease-infected plant debris was also spread in each plot to perpetuate and multiply the pathogen inoculum. Population dynamics of *Trichoderma viride*, *T. harzianum*, *Pseudomonas fluorescence*, *Bacillus subtilis*, and *Sclerotium rolfsii* were evaluated in rhizosphere soil by using the dilution plate count technique [29] (Hirte 1969). Rhizosphere soil samples were randomly taken from treated experimental groundnut field during 2017–2018. One gram soil was weighed and transferred to 10 mL of sterile distilled water, and then soil suspension was diluted from 10^1^ to 10^8^ after appropriately stirred. To estimate the population of *S. rolfsii*, *Trichoderma viride*, and *T. harzianum*, 1 mL of each soil suspension dilution was poured into the PDA-contained Petri plates with the help of a pipette. Tree replications of each dilution were kept. All the Petri plates were wrapped by parafilm and incubated at 28 ± 2 °C in BOD. The colony forming unit (CFU) of *Trichoderma viride*, *T. harzianum*, and *S. rolfsii* were observed in Petri plates after 3 days. The same technique was used for the estimation of the population of *Pseudomonas fluorescens* and *Bacillus subtilis*, but Nutrient agar media was used for the growth of bacteria using a dilution plate technique of rhizospheric soil at the dilutions of 10^1^ to 10^6^ and the colony was observed after 24 h in Petri plates.

Observation of disease incidence was recorded at the fortnightly interval after sowing. Disease incidence (DI) and Percent disease control (PDC) were calculated by using the following formulae: Disease incidence (DI) = [Number of infected plants/Total number of plants] × 100; Percent disease control (PDC) = [PDI in control − PDI in treatment/PDI in control] × 100; For the recording of dry weight, the groundnut plants were uprooted after 105 days of sowing, gently washed in tap water, and dried in the oven at 60 °C for 24 h. Five plants’ dry weight (DW) from each replication was recorded. Dry wt. in (%) was calculated using the following formula: Dry wt. in (%) = [Dry wt. in treatment − Dry wt. in control/Dry wt. of plants in control] × 100. Pod yield was calculated after harvesting groundnut. In observation of the weight of fresh pod yield, groundnut plants were pooled replication-wise individually after being harvested and dried, and then treatment-wise, mean weights of pod yield were calculated.

### 2.6. Statistical Analysis

Data on disease incidence in all the experiments were transformed into angular values before statistical analysis. Significant differences (*p* < 0.05) between the treatments were determined by Duncan’s multiple range test (DMRT). All analyses were done by using IBM SPSS v 16, Chicago, IL, USA).

## 3. Results

### 3.1. Isolation, Identification, and Pathogenicity of Sclerotium rolfsii

**Figure 3:**
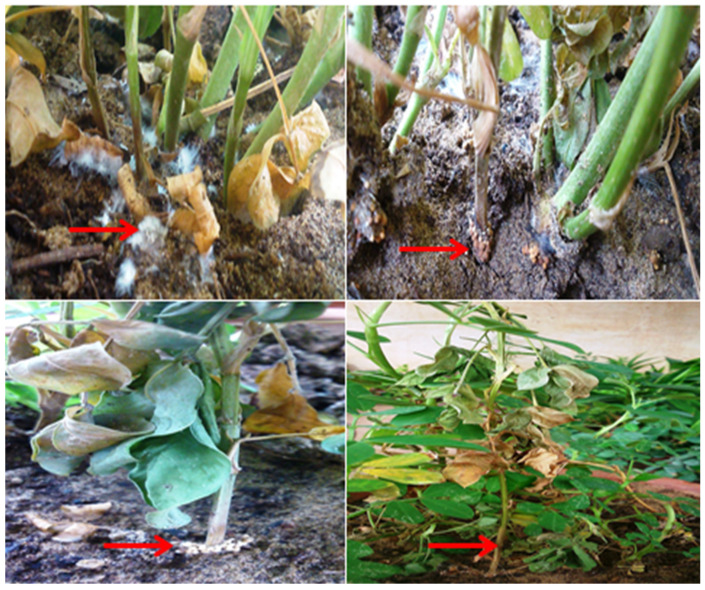
Validation of Koch’s postulate in groundnut plant.

Results revealed that infected stem pieces and sclerotia produced mycelium on PDA media. Initially, the mycelial growth was white; later, it produced immature whitish sclerotia, which became mature and changed from brown to dark brown (Figure 3). On maturity, sclerotium was very hard and old sclerotia showed a shiny appearance. The identification of fungus and Koch’s postulate was confirmed as described in the materials and methods section.

### 3.2. Antagonistic Activity of Bio-Agents against S. rolfsii under In Vitro Conditions

In the dual culture method, results revealed that maximum growth inhibition of the pathogen was achieved with *T. harzianum* (Th-BKN) (83.12%), followed by *T. viride* (Tv-BKN) (73.16%). Similarly, in the paper disc method, the results revealed that the *P. fluorescens* (Pf-BKN) was found to be relatively a greater inhibiter and gave maximum (41.55%) mycelial growth inhibition of *S. rolfsii*, followed by *B. subtilis* (Bs-BKN) (28.57%) after incubation.

The efficacy of different BCA culture filtrates, either alone or in combination, was also tested on the mycelial growth of *S. rolfsii* to ascertain their antagonistic activities. Nine treatments, including the control, were executed in 1, 2.5, and 5% concentrations on a PDA medium. Results from Table 2 revealed that the culture filtrate of the respective bio-agents reduced the mycelial growth of pathogens to various extents. Maximum mycelial percent growth inhibition was recorded with *T. harzianum* and *P. fluorescens* (79.61, 83.51, and 86.77%), followed by the treatment *T. viride* and *P. fluorescens* (75.74, 79.63, and 83.14%), while *B. subtilis* (Bs-BKN) (60.92, 65.36, and 69.92%) was found to be less inhibitory compared to the rest of the bio-agents. The media-amended culture filtrates of the combined bio-agents at 5% were found to be superior in reducing the growth of pathogens compared to culture filtrates of a single bio-agent.

### 3.3. Management of S. rolfsii by Bio-Agents under In Vivo Conditions

**Figure 4:**
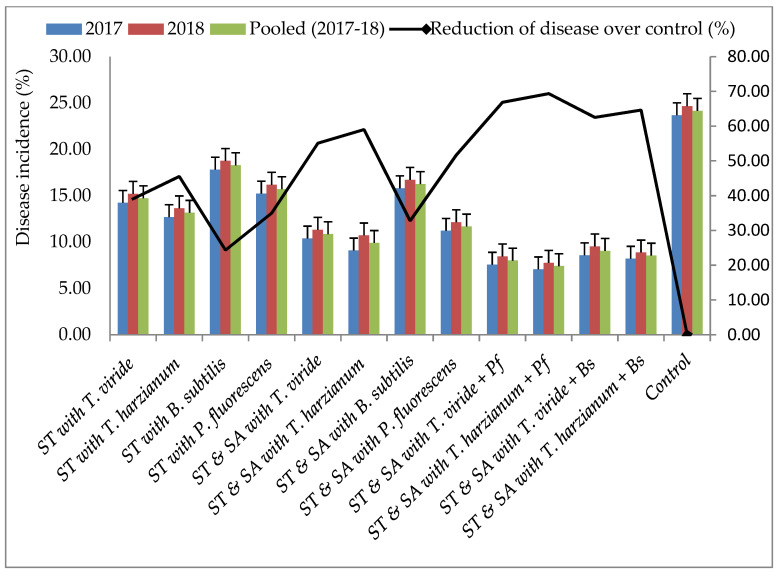
Effect of bio-agents on stem rot disease under field conditions; ST—Seed treatment; SA—Soil application.

**Figure 5:**
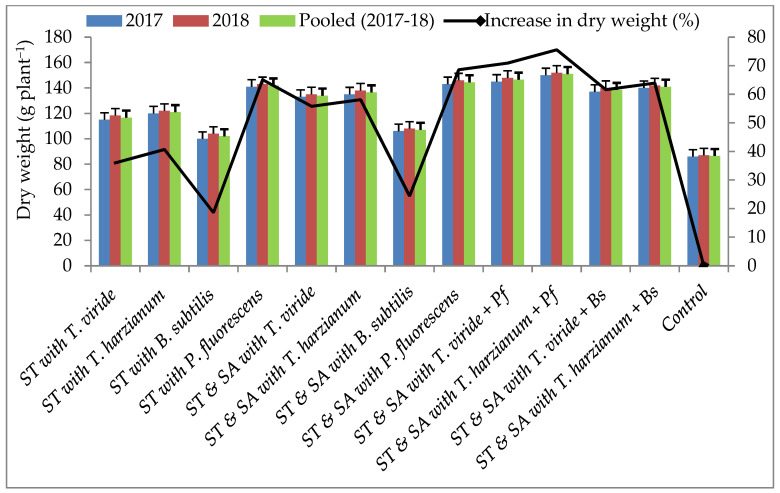
Effect of bio-agents on groundnut plants under field conditions; ST—Seed treatment; SA—Soil application.

**Figure 6:**
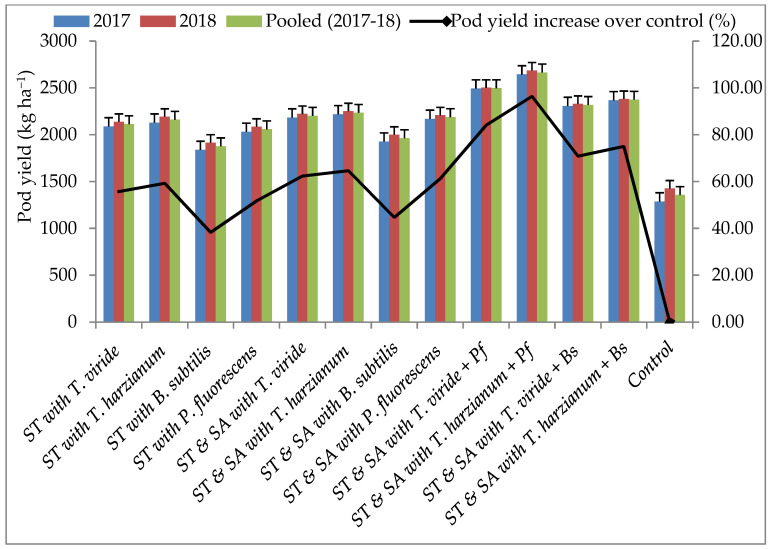
Effect of bio-agents on groundnut production under field conditions; ST—Seed treatment; SA—Soil application.

Within the pot experiments, bio-agents effectively suppressed the mycelial growth of the test pathogen. These potential bio-agents were also tested under field conditions. Results revealed that stem rot was effectively suppressed by seed treatment (ST) and soil application (SA) of the BCAs. The lowest disease incidence and highest disease control of stem rot (7.40 and 69.37%) were recorded in treatment T_10_—consisting of *T. harzianum* and *P. fluorescens*, followed by T_9_—consisting of *T. viride* and *P. fluorescens* (8.00 and 66.88%). In contrast, the highest disease and lowest disease control were observed (18.28 and 24.34%) in T_3_—consisting of the *B. subtilis* treatment (Figure 4). The highest DW and highest percent increase in DW was observed in T_10_—consisting of *T. harzianum* and *P. fluorescens* (151 g plant^−1^ and 75.58%), followed by T_9_—consisting of the *T. viride* and *P. fluorescens* (147 g plant^−1^ and 70.93%) treatment, whereas the lowest DW and lowest percent increase in DW was recorded in T_3_—consisting of *B. subtilis* (102 g plant^−1^ and 18.60%) over the control. However, all the bio-agent treatments positively influenced the DW of the plants (Figure 5). The highest pod yield (2665 and 2498 kg ha^−1^) and highest percent increase in pod yield (96.38 and 84.08%) were recorded in T_10_—consisting of *T. harzianum* and *P. fluorescens* and T_9_—consisting of *T. viride* and *P. fluorescens*, whereas the lowest pod yield and lowest percent increase in pod yield (1877 kg ha^−1^ and 38.32%) were recorded in T_3_—consisting of the *B. subtilis* treatment. All other BCA treatments also positively influenced pod yield compared to the control (Figure 6).

### 3.4. Inoculum Density of Microbial and Pathogen in Different Treatments

**Figure 7:**
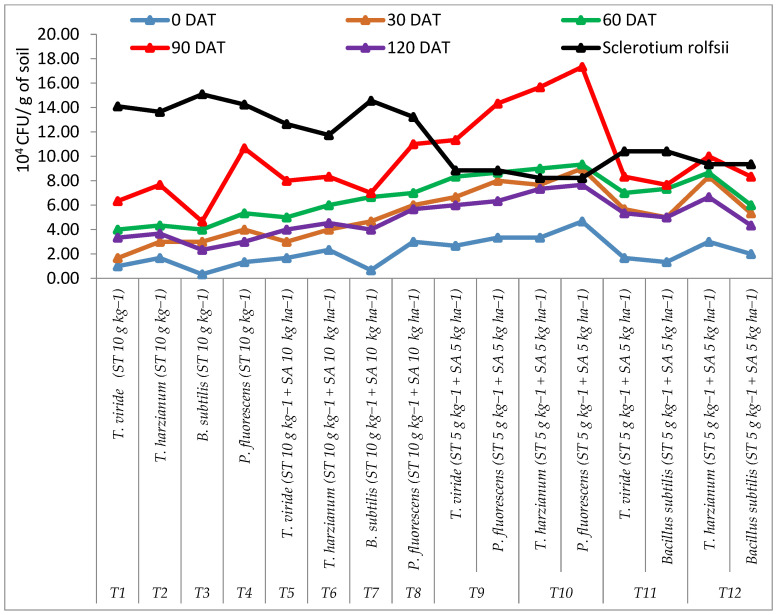
Rhizosphere average population of *Trichoderma viride*, *T. harzianum*, *Pseudomonas fluorescens*, *Bacillus subtilis*, and *Sclerotium rolfsii* in different treatments during 2017–2018.

Results revealed that the average population of the BCA (*T. viride*, *T. harzianum*, *P. fluorescence*, and *B. subtilis*) increased up to 90 DAT in all treatments; thereafter, the populations of all BCAs were decreased. The average population of *T. viride*, *T. harzianum*, *P. fluorescens*, and *B. subtilis* was recorded from zero DAT to 120 DAT from rhizosphere soil during 2017–2018. The average population of *T. viride* was recorded in the range of 1.0 × 10^4^ to 11.33 × 10^4^ cfu/g, while the average population of *T. harzianum* was observed in the range of 1.67 × 10^4^ to 15.67 × 10^4^ cfu/g. Similarly, the average population of *P. fluorescens* was recorded in the range of 1.33 × 10^4^ to 17.33 × 10^4^ cfu/g, while the average population of *B. subtilis* was observed in the range of 0.33 × 10^4^ to 8.33 × 10^4^ cfu/g. Maximum average cfu/g soil for *T. harzianum* and *P. fluorescens* (15.67 × 10^4^ and 17.33 × 10^4^ cfu/g) was recorded at 90 DAT, followed by 9.00 × 10^4^ and 9.33 × 10^4^ cfu/g at 60 DAT, 7.67 × 10^4^ and 9.00 × 10^4^ cfu/g at 30 DAT, and 3.33 × 10^4^ and 4.67 × 10^4^ cfu/g at zero DAT in treatment, consisting of seed treatment and soil application with *T. harzianum* and *Pf* at 10 (5 + 5) g kg^−1^ + 10 (5 + 5) kg ha^−1^ compared to the control (1.33 × 10^4^ and 1.67 × 10^4^ cfu/g) during the year of 2017–2018. Similarly, the maximum average populations of *T. viride* and *B. subtilis* (8.33 × 10^4^ and 7.67 × 10^4^ cfu/g) was recorded at 90 DAT, followed by 7.00 × 10^4^ and 7.33 × 10^4^ cfu/g at 60 DAT, 5.67 × 10^4^ and 5.00 × 10^4^ cfu/g at 30 DAT, and 1.67 × 10^4^ and 1.33 × 10^4^ cfu/g at zero DAT in treatment, consisting of seed treatment and soil application with *T. viride* and *Bs* at 10 (5 + 5) g kg^−1^ + 10 (5 + 5) kg ha^−1^ compared to the control (1.00 × 10^4^ and 0.33 × 10^4^ cfu/g). The lowest average population of *T. viride*, *T. harzianum*, *P. fluorescens*, and *B. subtilis* (6.0 × 10^4^, 7.00 × 10^4^, 7.67 × 10^4^, and 5.0 × 10^4^ cfu/g) was also observed at 120 DAT, respectively. The lowest average population of *S. rolfsii* (8.23 × 10^4^ cfu/g) was observed in treatment consisting of seed treatment and soil application with *T. harzianum* and *Pf* at 10 (5 + 5) g kg^−1^ + 10 (5 + 5) kg ha^−1^, followed by 8.85 × 10^4^ cfu/g in treatment consisting of seed treatment and soil application with *T. viride* and *Pf* at 10 (5 + 5) g kg^−1^ + 10 (5 + 5) kg ha^−1^, respectively. While the highest average population (15.09 × 10^4^ cfu/g) was recorded in treatment consisting of seed treatment with *B. subtilis* at 10 g kg^−1^ (Figure 7).

## 4. Discussion

### 4.1. Antagonistic Activity of Bio-Agents against S. rolfsii under In Vitro Conditions

Due to the soil-borne nature of *Sclerotium rolfsii*, the application of chemicals is hardly successful in the presence of a high level of inoculum in the soil and favorable climatic factors. Therefore, a cost-effective and eco-friendly approach would be the use of a biological control. The present study focused on the management of *S. rolfsii* with possible fungal and bacterial bio-agents. The antagonistic potential of BCAs against *S. rolfsii* was determined using the dual culture, paper disc, and culture filtrate methods. All bioagents efficiently inhibited the growth of *S. rolfsii*. During the study, we assessed the potentiality of fungal and bacterial bio-agents, both singly and in combinations, and it was observed that the combined formulation of *T. harzianum* and *P. fluorescens* suppressed the maximum percent growth inhibition of the *S. rolfsii* pathogen, followed by *T. viride* and *P. fluorescens*. Both BCAs developed faster than *S. rolfsii*, indicating that pathogenic fungi and antagonists competed for space and nutrients. It can be speculated that the activity of different hydrolytic enzymes released by the bio-agents caused this inhibition zone, which revealed that *T. harzianum* was superior in controlling the test pathogen compared to *T. viride*. The synthesis of hydrolytic enzymes (cellulases, chitinases, glucanases, and proteases) and secondary metabolites against *S. rolfsii* was observed by Sharma (2011) and Nicolas (2014) [30,31]. Prajapati et al. 2015 [32] reported that *T. harzianum* and *T. viride* completely inhibited mycelial development and sclerotia generation in *S. rolfsii* after 4–8 days of incubation. Similarly, Shivakumar et al. 2020 [33] observed that among the 10 isolates of *T. viride* and *P. fluorescens*, the isolates Tv3 and Pf5 effectively inhibited the mycelial growth and sclerotia of *S. rolfsii* under in vitro conditions. According to Subhendu Josh and Pan (2004) [34], a 2% concentration of the *T. harzianum* culture filtrate hindered the maximal mycelial growth of *R. solani*. These findings are also consistent with our studies.

*P. fluorescens* (Pf-BKN) was determined to be the most efficient bacterial bio-agent in terms of mycelial growth suppression, followed by *B. subtilis*. Both bacterial bio-agents considerably inhibited mycelial growth and were deemed superior. Babu and Kumar 2008, Rakh et al. 2011, and Darvin and Prasanna 2013 [35,36,37] observed that *T. harzianum* was more efficient against the mycelial growth of *S. rolfsii* than *T. viride*, *Pseudomonas fluorescens*, and *B. subtilis*. Suneeta et al. 2016 [38] reported that 26 *Bacillus* spp. isolates were tested in vitro against the collar rot pathogen. Five *Bacillus* spp. strains demonstrated the highest antagonistic activity against *S. rolfsii*. Similarly, Rajendra Prasad et al. 2017 [39] tested the efficiency of 24 fungal biocontrol agents and 12 bacterial biocontrol agents against the phytopathogenic fungus *Sclerotium rolfsii* using a dual culture approach. The BCAs *T. harzianum*-1 and *P. f*-3 were shown to be the most effective at inhibiting mycelium against *Sclerotium rolfsii* in vitro (95.1 and 60.80%), respectively. These findings are also consistent with our results. The BCAS *T. harzianum* and *T. viride* were shown to be significantly effective due to their ability to colonize the hyphae, disrupt the mycelial growth by producing volatile metabolites, and kill the pathogen [40,41]. Similarly, *P. fluorescens* was also found effective due to their excellent root colonizing ability and production of antifungal metabolites like pyoluteorin, 2,4- diacetyl phloroglucinol (DAPG), pyrrolnitrin, and phenazines against the hyphae of *S. rolfsii* [42,43], but it may be a possibility that *B. subtilis* was less inhibiting due to competition for food with other microbials. However, lower concentrations had a lower inhibitory effect on *S. rolfsii* growth. The 5% culture filtrate of the bioagents was shown to be more effective and superior than the 1.0% and 2.5% concentrations. During the investigation, we also observed that the mixed culture filtrate of the BCA was more efficient against test pathogens than separate BCA culture filtrates. Darvin et al. 2013 [37] revealed that the *T. viride* culture filtrate (Tv L) was found to be the most effective, inhibiting *S. rolfsii* to the greatest extent, followed by *T. harzianum* 14 (Th14) and *T. harzianum* (Th4). Similarly, Mishra et al. 2011 [44] found that the culture filtrate of *T. viride* (Tr 8) was effective against *M. phaseolina* and other soil-borne pathogens, which supports our results.

### 4.2. Management of S. rolfsii by Bio-Agents under Field Conditions

Groundnut stem rot is a major disease in most groundnut growing areas and often causes considerable pod yield losses if the crop is cultivated continuously in the same field for over two to three seasons. Due to the soil-borne nature of *S. rolfsii* and favored climatic conditions, it spreads very fast on the host crop. So, the antagonistic activities of bio-agents evaluated under in vitro and pots conditions were also tested under field conditions. Talc-based bio-agent formulations of *T. harzianum* and *P. fluorescens* and *T. viride* and *P. fluorescens* used in seed and soil treatment significantly reduced disease incidence. The BCAs *T. harzianum* and *P. fluorescens* and *T. viride* and *P. fluorescens* reduced the disease and increased plant growth. In addition, we observed that dual formulations of BCAs were more effective against *S. rolfsii* than individual BCAs. Manjula et al. 2004 [45] found that combining seed treatment with *P. fluorescens* (Pf1) (seed soaked in cell solution 10^9^ CFU ml^−1^ for 5 min) with coated seed treatment with *T. viride* (2 g kg^−1^) greatly reduced the occurrence of stem rot. The maximum average cfu/g soil for *T. harzianum* and *P. fluorescens* (15.67 × 10^4^ and 17.33 × 10^4^ cfu/g) was recorded in combined talc-based formulations which reduced the population density of *S. rolfsii*. The population dynamic of bioagents at zero days was very low but later on it increased rapidly and peaked at 90 days. This finding is corroborated by studies conducted by [46,47]. Doley et al. 2017 [48] reported that the incidence of stem rot was found to be 45.83% in the combined application of mycorrhiza and *Trichoderma* (Gf+Sr+Tv) in the presence of the pathogen, as compared to 54.17% in the single application of mycorrhiza (Gf+Sr), 59.72% *Trichoderma* (C+Sr+Tv), and 68.06% in control ones (C+*S. rolfsii*). Similarly, Meena et al. 2018 [49] found that single and combined bio-agent treatments effectively control *S. rolfsii*. We observed that the application of farm yard manure (FYM) in experimental plots increased the multiplication and potentiality of bio-agents because FYM retains moisture, maintains the EC and pH, increases aeration in soil, and provides carbon and congenial environmental conditions to bio-agents and other beneficial microorganisms. Moreover, a BCA that may persist in the rhizosphere for years could be an important advantage over applying chemical pesticides. Jadon et al. 2018 [50] reported that the lowest percent stem rot was recorded in the soil application of *Trichoderma* isolate T- 170 enriched in FYM at 1 ton ha (50 kg per 200 kg FYM) and seed treatment of *Pseudomonas fluorescens* at 10 mL kg seed (TNAU) and seed treatment of *Pseudomonas fluorescens* at 10 mL kg seed (DGR) (T13). Similarly, Safari Motlagh et al. 2022 [14] observed that *T. viride*, *A. flavus*, and *P. rubens* were 44%, 42%, and 38% effective in alleviating the disease incidence and severity and positively increased the height, fresh weight, and dry weight of the groundnut plants, which also corroborates our study.

The dry weight and pod yield of plants were also substantially increased and considerably higher in the combined treatment of *T. harzianum* and *P. fluorescens*, followed by *T. viride* and *P. fluorescens*. Similarly, the highest increased DW was observed with the same BCA treatments. The BCA *P. fluorescens*, individually or with a combination of other bio-agents, also enhanced the growth of plants due to PGPR activities. This treatment module considerably reduced groundnut seedling mortality compared to the untreated control. In a similar line of research, Rakholiya and Jadeja 2010 [51] evaluated various bio-agents with seed treatment for the management of stem rot of groundnut under field conditions and found significantly less disease incidence and high yield with seed treatment of *T. harzianum* (10 g kg^−1^). Similarly, Rasu et al. 2013 [52] reported that the combined soil application of farmyard manure-based bio-formulation of *P. fluorescens* (Pf1) (2.5 g/pot) and *T. asperellum* (Th1) (2.5 g/pot) resulted in significantly less incidence of root rot of sugar beet caused by *S. rolfsii*. Sarita et al. 2018 [53] also reported the effectiveness of *T. harzianum* and FYM, which gave a maximum reduction of stem rot, followed by *T. harzianum*, which agrees with our study. Seed treated with *T. harzianium* (4 g kg^−1^ seed) and carboxin (0.5 g kg^−1^ seed) provided maximum protection to the chickpea crop by giving maximum seedling emergence (495.0/20 m^2^), final plant stand (480/20 m^2^), and grain yield (l8.2 q ha^−1^), according to Kumar et al. 2008 [54]. Rajendra Prasad et al. 2017 [39] tested *Pseudomonas fluorescence* and *Trichoderma harzianum* in several treatment combinations against tomato *S. rolfsii*. They found that seed treatment with *Pseudomonas fluorescence*-3 and soil application with *Trichoderma harzianum*-1, followed by seed treatment with *Trichoderma harzianum*-1 and soil application with *Pseudomonas fluorescence*-3, resulted in 52.08 and 49.17% germination when inoculated with *S. rolfsii*, respectively, which supports our findings.

## 5. Conclusions

In the present study, the research results performed under in vitro and in vivo conditions revealed that the groundnut stem rot disease can significantly be controlled by the BCAs *T. viride*, *T. harzianum*, *P. fluorescens*, and *Bacillus subtilis*. Based on our results, it is concluded that the dual formulation of *T. harzianum* and *P. fluorescens*, followed by *T. viride* and *P. fluorescens*, were the most effective BCAs in suppressing the *S. rolfsii* and enhancing the dry weight and pod yield under in vitro and in vivo conditions. Therefore, an integrated disease management module with these BCAs needs to be developed and validated with a farmers’ participatory mode under field conditions.

## Figures and Tables

**Table 1:** Detail of bio-agents used under field conditions.

Treatment	Details of Treatments
T_1_	Seed treatment with *Trichoderma viride* at 10 g kg^−1^
T_2_	Seed treatment with *Trichoderma harzianum* at 10 g kg^−1^
T_3_	Seed treatment with *Bacillus subtilis* at 10 g kg^−1^
T_4_	Seed treatment with *Psuedomonas fluorescens* at 10 g kg^−1^
T_5_	Seed treatment and soil application with *T. viride* at 10 g kg^−1^ + 10 kg ha^−1^
T_6_	Seed treatment and soil application with *T. harzianum* at 10 g kg^−1^ + 10 kg ha^−1^
T_7_	Seed treatment and soil application with *Bacillus subtilis* at 10 g kg^−1^ + 10 kg ha^−1^
T_8_	Seed treatment and soil application with *P. fluorescens* at 10 g kg^−1^ + 10 kg ha^−1^
T_9_	Seed treatment and soil application with *T. viride* + *Pf* at 10 (5 + 5) g kg^−1^ + 10 (5 + 5) kg ha^−1^
T_10_	Seed treatment and soil application with *T. harzianum* + *Pf* at 10 (5 + 5) g kg^−1^ + 10 (5 + 5) kg ha^−1^
T_11_	Seed treatment and soil application with *T. viride* + *Bs* at 10 (5 + 5) g kg^−1^ + 10 (5 + 5) kg ha^−1^
T_12_	Seed treatment and soil application with *T. harzianum* + *Bs* at 10 (5 + 5) g kg^−1^ + 10 (5 + 5) kg ha^−1^
T_13_	Control (treated with distilled water)

**Table 2:** Effect of bioagents on *Sclerotium rolfsii*.

Treatment	1%		2.5%		5%	
	Mycelial Growth (mm) **	Inhibition (%)	Mycelial Growth (mm) **	Inhibition (%)	Mycelial Growth (mm) **	Inhibition (%)
*T. viride*	31.68 ± 0.78 (34.25) *	64.80	27.83 ± 0.76 (31.84) *	69.07	24.17 ± 0.76 (29.45) *	73.14
*T. harzianum*	25.83 ± 0.29 (30.55)	71.30	21.67 ± 0.29 (27.74)	75.92	18.83 ± 0.29 (25.72)	79.05
*B. subtilis*	35.17 ± 1.04 (36.37)	60.92	31.17 ± 0.30 (33.94)	65.36	27.07 ± 0.12 (31.35)	69.92
*P. fluorescens*	32.33 ± 1.53 (34.65)	64.07	29.00 ± 0.25 (32.58)	67.77	24.33 ± 0.58 (29.56)	72.96
*T. viride* + *P. fluorescens*	21.83 ± 1.75 (27.86)	75.74	18.33 ± 0.27 (25.35)	79.63	15.17 ± 0.76 (22.92)	83.14
*T. harzianum* + *P. fluorescens*	18.31 ± 0.35 (25.35)	79.61	14.84 ± 0.23 (22.66)	83.51	11.90 ± 0.08 (20.18)	86.77
*T. viride* + *Bacillus subtilis*	26.67 ± 2.08 (31.09)	70.37	22.50 ± 0.50 (28.32)	75.00	20.33 ± 0.58 (26.80)	77.41
*T. harzianum* + *Bacillus subtilis*	23.50 ± 0.50 (29.00)	73.88	18.67 ± 0.76 (25.60)	79.25	16.50 ± 0.57 (23.97)	81.66
Control (without culture filtrate)	90.00 ± 1.12 (71.57)	-	90.00 ± 31.22 (71.57)	-	90.00 ± 1.41 (71.57)	-
S.Em (±)	0.43	0.73	(0.18)	0.30	(0.23)	0.37
C.D (*p* = 0.05)	1.27	2.19	(0.53)	0.91	(0.69)	1.12
C.V (%)	2.07	1.80	(0.93)	0.70	(1.28)	0.82

* Figures in parentheses are angular transformed values; ** Mean of three replications.

## Data Availability

The datasets generated during and/or analyzed during the current study are available from the corresponding author on reasonable request.
